# Serum microRNA‐126 Levels Are Associated With Diabetic Nephropathy in Patients With Type 2 Diabetes Mellitus

**DOI:** 10.1155/ije/6277857

**Published:** 2026-01-02

**Authors:** Jie Yun, Tianqi Liu, Yake Lan, Chaofan Dong, Xin Bao, Liming Zhu, Shan Luo, Liqun Song, Yexu Song

**Affiliations:** ^1^ Department of Nephrology, First Affiliated Hospital of Heilongjiang University of Chinese Medicine, Harbin, 150040, China, hljucm.edu.cn; ^2^ Department of General Surgery, Third Affiliated Hospital of Heilongjiang University of Chinese Medicine, Harbin, 150000, China; ^3^ Department of Graduate School, Heilongjiang University of Chinese Medicine, Harbin, 150040, China, hljucm.edu.cn; ^4^ Department of Science and Technology, Heilongjiang University of Chinese Medicine, Harbin, 150004, China, hljucm.edu.cn

**Keywords:** biomarker, diabetic nephropathy, microRNA, prognosis, Type 2 diabetes mellitus

## Abstract

**Objective:**

Diabetic nephropathy (DN) occurs in nearly 40% of Type 2 diabetes mellitus (T2DM) patients, and there is a positive correlation between DN and terminal renal disease. Thus, exploring novel biomarkers is vital to facilitate early diagnosis and intervention in DN patients; however, indicators of DN are still scant. Since the microRNA‐126 (miR‐126) may be related to the occurrence of diabetes, we aim to assess the association between miR‐126 and DN.

**Methods:**

This is a prospective cohort design and a nested case–control approach study that enrolled 300 individuals (100 T2DM patients, 100 DN patients, and 100 controls). miR‐126 expression was analyzed by quantitative real‐time PCR (qPCR) and compared among three groups. The overall survival (OS) was presented by Kaplan–Meier analysis. The area under the curve (AUC) was used to evaluate the potential of miR‐126 as a biomarker for DN.

**Result:**

DN patients, compared with T2DM and controls, had lower miR‐126 content (*p* < 0.001), and miR‐126 levels significantly decreased following a decreasing estimated glomerular filtration rate (eGFR) (*r* = 0.65, *p* < 0.001). Moreover, significant differences were also found in OS among quartiles of serum miR‐126 level (*p* for trend < 0.001). In addition, the AUC for diagnosis DN from T2DM patients was found to be 0.804 (95% CI, 0.745–0.863), with a sensitivity of 83.0% and a specificity of 63.0%.

**Conclusion:**

This study provides evidence to clarify that decreased levels of miR‐126 were linked to an increased susceptibility to developing DN compared with healthy volunteers. More importantly, the diagnostic role of miR‐126 remained significant in differentiating DN from T2DM patients.

## 1. Introduction

Diabetes mellitus (DM) has been regarded as a chronic metabolic disorder featuring a deficiency in insulin secretion and action [1]. Among all DM‐related comorbidities, diabetic nephropathy (DN) was considered one of the leading causes of end‐stage renal disease (ESRD) [2]. About 40% of DM patients are complicated by DN, which further results in increased morbidity and mortality in DM patients. In China, DN imposes a heavy burden, which has the largest number of people with diabetes all over the world [3]. In 2017, over 20% of dialysis patients had diabetes in China [4]. Furthermore, the all‐age mortality owing to DN in China increased by 33.3% [5]. These challenges underscore the imperative of understanding the DN to curb its epidemic.

Glucose metabolism disorder, oxidative stress, and inflammation have been considered the major causes of DN, but the potential mechanism is still unclear [6]. According to a previous study, the prevalence of hypertension and electrolyte disturbances was significantly higher in the type 2 diabetes mellitus (T2DM) with the DN group. Additionally, these factors may also promote the progression of T2DM to DN [7]. Generally, the diagnosis of DN and its severity are based on histological changes based on the biopsy sample of the kidney and laboratory indices such as glomerular filtration rate (GFR), albuminuria, and proteinuria. However, a kidney biopsy is an invasive procedure that may not be applicable to some patients [8]. Moreover, there were limitations in utilizing albuminuria as an indicator of DN, allowing for some patients to experience GFR deterioration without progress in albuminuria [9]. Thus, it is vital to seek a noninvasive biomarker to reflect the pathogenesis of DN, which may allow earlier diagnosis and stratify the severity of DN.

To date, emerging research has discovered that circulating microRNAs (miRs) are associated with DM, and the key role of microRNAs in DN has received increasing attention from researchers [10]. miR‐126 was originally reported to be implicated in several kinds of diseases, and its dysfunction can lead to profound impairment of glucose metabolism [11–13]. These studies provide evidence to suggest that miR‐126 expression is related to the regulation of glucose homeostasis. DN can be considered the most common microvascular complication in patients with diabetes. MiR‐126 is reported to be downregulated in DM patients with microvascular complications, leading to increased neovascularization via VEGF/MAPK pathway activation [14]. Moreover, a previous study had indicated the therapeutic potential of enhanced expression of miRNA‐126 that targeted oxidative stress in T2DM patients [15].

However, the diagnostic role of miR‐126 in T2DM patients who suffered the DN remains unclear. Thus, we aim to explore the diagnostic value of miR‐126 in the occurrence of DN and their association with diabetes risk factors and markers of kidney injury.

## 2. Materials and Methods

### 2.1. Study Cohort

This is a prospective cohort design and a nested case–control approach study that enrolled 300 consecutive persons, who were recruited from the Department of Nephrology, First Affiliated Hospital of Heilongjiang University of Chinese Medicine, from January 2016 to December 2021. These patients were divided into three groups: T2DM with DN group (DN group), T2DM without DN group (DM group), and the control group (people with normal glucose tolerance). The median follow‐up time was 42 (14–60) months. We excluded patients with Type 1 DM or advanced chronic diabetes complications. Besides, participants with liver damage, rheumatic immune system diseases, cancer, cardiovascular disease, and nephropathy or nephritis caused by other diseases were excluded from this study. The follow‐up time begins on the date of admission and ends on the date of death or the last follow‐up, respectively.

Normal glucose tolerance was evaluated by an oral glucose tolerance test (OGTT) with frequent sampling for the determination of plasma glucose as previously stated [16, 17]. T2DM was defined based on fasting plasma glucose (FPG) concentration of ≥ 200 mg/dL or the provision of pharmacological treatment [18].

The presence of DN was defined as the occurrence of at least two consecutive measurements of albumin‐to‐creatinine ratio exceeding 2.5 mg/mmol for men and 3.5 mg/mmol for women or by a single instance of albuminuria detection while the patient was undergoing treatment with an angiotensin‐II‐receptor antagonist [19, 20].

Patient characteristics and risk factors were collected at admission, including age, gender, body mass index (BMI), and medical history (hypertension and smoking status). Use of statins, oral glucose–lowering drugs, and insulin was also documented. Biochemistry detection includes FBG, glycated hemoglobin (HbA1c), creatinine (CR), uric acid (UA), and estimated glomerular filtration rate (eGFR). Moreover, low‐density lipoprotein cholesterol (LDL‐C), high‐density lipoprotein cholesterol (HDL‐C), total cholesterol (TC), and triglyceride (TG) were also collected. eGFR was assessed by the creatinine‐based Chronic Kidney Disease Epidemiology Collaboration (CKD‐EPI) equation [21].

The study was approved by the Ethics Committee of the First Affiliated Hospital of Heilongjiang University of Chinese Medicine, and all participants signed the informed consent before enrollment.

### 2.2. Laboratory Measurements

Blood samples (5 mL) were collected after admission and then transferred to EDTA and citrate blood collection tubes. Total RNA was extracted from 0.3 mL of serum by TRIzol LS reagent (Invitrogen, Carlsbad, USA). The miR‐126 level was assessed by quantitative polymerase chain reaction (qPCR). The cDNA obtained was amplified using the specific TaqMan microRNA assays (Life Technologies).

Then, qPCR was performed using an ABI Prism 7500 sequence detection system (Applied Biosystems). The amplification reactions were incubated at 95°C for 30 min, followed by 40 cycles at 94°C for 15 s, 55°C for 30 s, and 70°C for 30 s. The expression level of miR‐126 was quantified in accordance with the cycle threshold (Ct) method.

### 2.3. Statistical Analysis

Data were statistically analyzed using SPSS (ver.22.0; SPSS Incorporated, Chicago, IL, USA) and GraphPad Prism 8.00 software. Skewed distribution data were expressed as median and range and performed using the Mann–Whitney *U* test and Kruskal–Wallis *H* test. Categorical variables were described with percentages and compared between the groups using the *χ* ^2^ test. The relevance of miR‐126 expression with clinical indicators, such as eGFR and HbA1c, was analyzed with Spearman’s rank correlation. The area under the receiver operating characteristic (AUROC) curve was also computed to assess the potential of miR‐126 as a biomarker for DN patients. Major adverse cardiovascular and cerebrovascular events (MACCE) were defined as angina, acute myocardial infarction, cardiac arrest, arrhythmia, heart failure, stroke, in‐hospital all‐cause mortality, or any combination of them. The sample size was determined assuming an expected probability of exposure rate of 25% in the control group and an odds ratio of 3.0. With *α* = 0.05, power = 90%, and a 1:1:1 case–control ratio, 70 DN cases, 70 T2DM cases, and 70 controls were required. Thus, this study included 300 participants (100 nondiabetic health volunteers and 100 T2DM patients and 100 DN patients), which can meet the needs of this research. The MACCE was estimated using the Kaplan–Meier method, and survival estimates were compared using the log‐rank test. *p* values < 0.05 were considered statistically significant.

## 3. Results

### 3.1. Baseline Characteristics

A total of 100 nondiabetic health volunteers and 200 participants were recruited, comprising 100 T2DM patients and 100 DN patients, and the median follow‐up time was 42 (IQR: 14–60) months. The baseline characteristics are shown in Table 1; of all included patients, 139 (46.3%) and 110 (36.7%) patients were current smokers. No differences were witnessed in smoking status, history of hypertension, HDL‐C, LDL‐C, TC, TG, gender, and age in all three groups (all *p* > 0.05), while a marked difference was observed in Cr, eGFR, and HbA1c (all *p* < 0.05).

**Table 1:** Baseline characteristics of the study population.

	Healthy control (*N* = 100)	T2DM (*N* = 100)	DN (*N* = 100)	*p* value
Male, no. (%)	61 (61.0)	65 (65.0)	70 (70.0)	0.407
Age (years), median (range)	60 (40–86)	59 (49–74)	61 (40–87)	0.960
eGFR (mL/min/1.73 m^2^), median (range)	119 (63–159)	96 (61–132)	69 (36–112)	< 0.001
HbA1c (%)	5.40 (4.54–5.88)	10.44 (7.79–13.84)	11.41 (8.14–14.27)	< 0.001
Cr, μmol/L, median (range)	48.4 (35.1–65.0)	61.1 (48.0–74.7)	93 (70–267)	< 0.001
Smoker or former smoker, no. (%)	38 (38.0)	36 (36.0)	36 (36.0)	0.944
Hypertension, no. (%)	42 (42.0)	47 (42.0)	50 (50.0)	0.518
Oral glucose‐lowering drugs, no. (%)	—	62 (62.0)	64 (64.0)	0.770
Insulin use, no. (%)	—	12 (12.0)	15 (15.0)	0.535
Statin use, no. (%)	34 (34.0)	45 (45.0)	44 (44.0)	0.217
HDL‐C, mmol/L, median (range)	1.17 (0.57–4.30)	1.18 (0.57–5.20)	1.25 (0.71–5.2)	0.864
LDL‐C, mmol/L, median (range)	1.95 (0.57–4.30)	1.98 (0.81–4.26)	2.13 (0.84–4.32)	0.101
TG, mmol/L, median (range)	1.18 (0.42–3.86)	1.17 (0.42–3.86)	1.17 (0.49–3.95)	0.698
TC, mmol/L, median (range)	3.28 (1.95–6.03)	3.55 (1.93–6.95)	3.60 (1.93–6.95)	0.390

Abbreviations: CR = creatinine, DN = diabetic nephropathy, HDL‐C = high‐density lipoprotein cholesterol, LDL‐C = low‐density lipoprotein cholesterol, TC = total cholesterol, TG = triglyceride.

### 3.2. Stratified Analysis of miR‐126 Among Groups and Correlation Analysis Related to Clinical and Pathological Parameters

**Figure 3:**
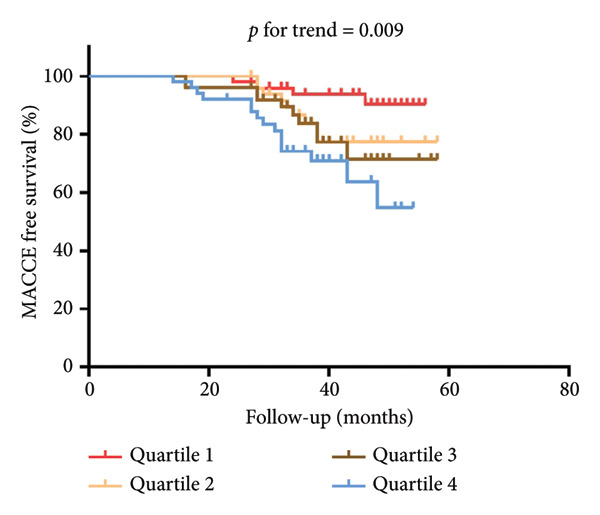
The Kaplan–Meier survival curve for the main adverse cardiovascular and cerebrovascular events (MACCEs) free survival distribution in all participants according to quartiles of circulating miR‐126 levels.

Serum miR‐126 levels were numerically lower in DN patients and DM patients than in healthy controls (all *p* < 0.001); moreover, there was also a statistically significant difference between the DN and DM groups (*p* < 0.001) (Figure 1(a)). Additionally, we also observed a lower serum miR‐126 level in patients with abnormal HbA1c ≥ 6%, compared with those with normal HbA1c levels (Figure 1(b)). Figure 2(a) illustrates the relevance of HbA1c with miR‐126 content, and the results indicate that the miR‐126 level harbored a negative relevance to HbA1c (*r* = −0.53, *p* < 0.001), while Figure 2(b) shows that eGFR positively associated with miR‐126 level (*r* = 0.65, *p* < 0.001). Kaplan–Meier survival analysis revealed statistically significant differences in MACCE‐free survival distributions among quartiles of serum miR‐126 level (Figure 3, log‐rank test, *p* for trend < 0.001).

Figure 1(a) Expression of circulating miR‐126 among diabetic nephropathy (DN) patients, type 2 diabetes mellitus (T2DM) patients, and healthy controls. (b) Association of expression of circulating miR‐126 with the glycated hemoglobin (HbA1c) levels.

**Figure:**
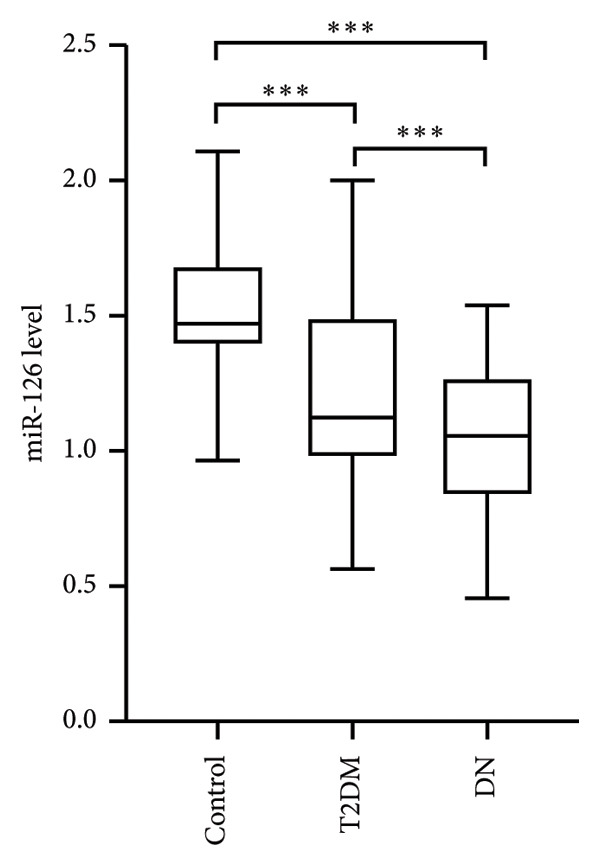
(a)

**Figure:**
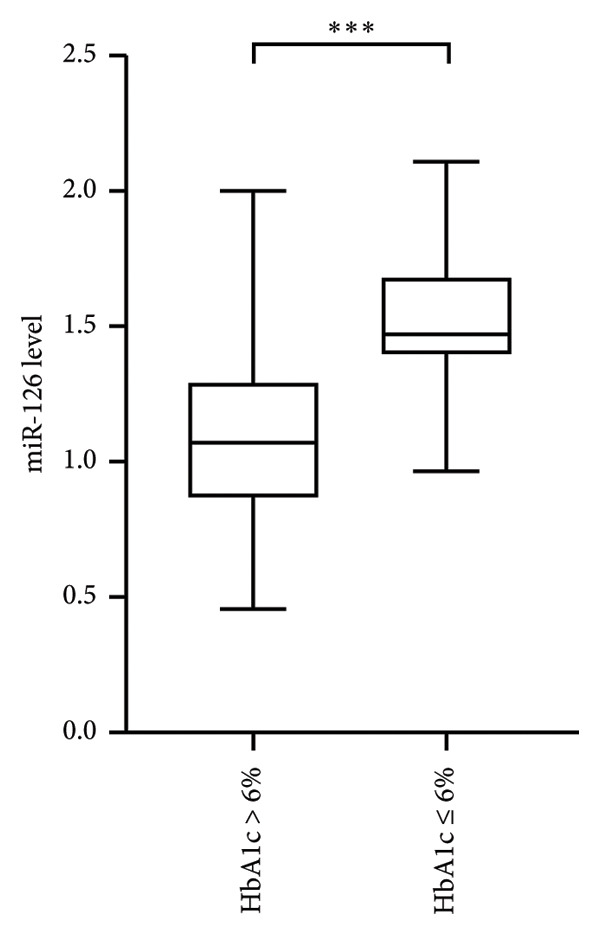
(b)

Figure 2(a) The Spearman correlation analysis of circulating miR‐126 levels with the concentration of glycated hemoglobin (HbA1c). (b) The Spearman correlation analysis of circulating miR‐126 levels with the estimated glomerular filtration rate (eGFR).

**Figure:**
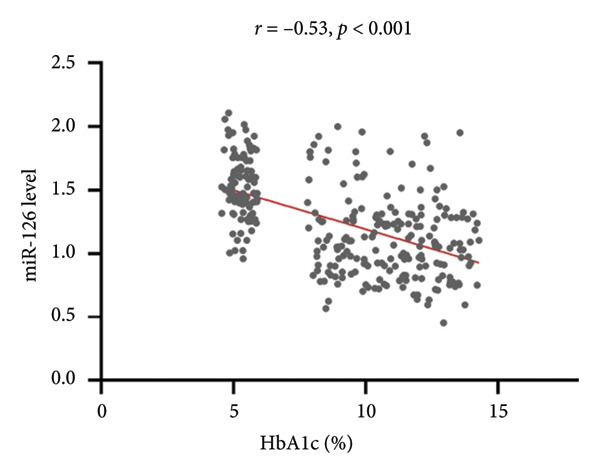
(a)

**Figure:**
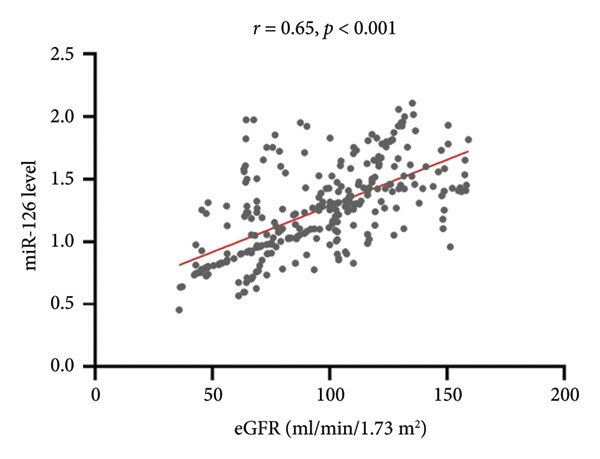
(b)

### 3.3. Diagnostic Potential of miR‐126 Level in Patients With DN

ROC analysis was conducted to examine the potential of miR‐126 as a biomarker for patients with DM from healthy controls (Figure 4(a)). The area under the curve (AUC) was 0.872 (95% CI, 0.831–0.913; *p* < 0.001). The sensitivity and specificity of miR‐126 in differentiating DM patients from healthy controls is 91.0% and 81.0%. More importantly, miR‐126 also had a favorable diagnostic power for DN from DM patients (AUC: 0.804 and 95% CI: 0.745–0.863) with a sensitivity of 83.0% and a specificity of 63.0% (Figure 4(b)).

Figure 4(a) ROC analysis of miR‐126 in identifying patients with type 2 diabetes mellitus (T2DM) from health controls. (b) ROC analysis of miR‐126 in identifying patients with T2DM from diabetic nephropathy (DN) patients.

**Figure:**
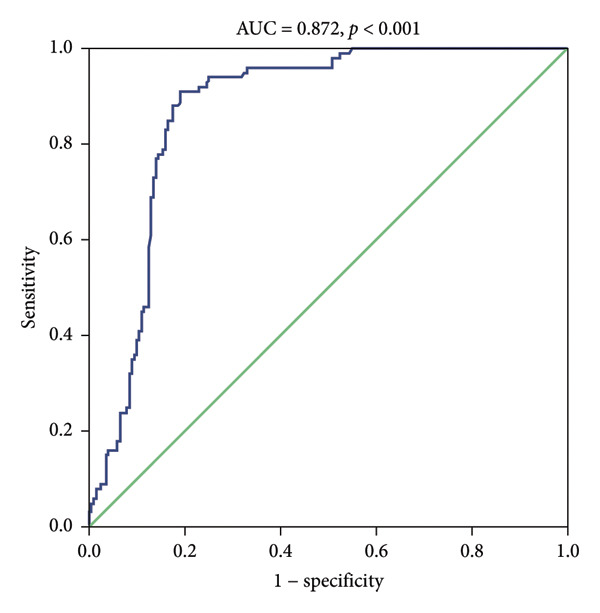
(a)

**Figure:**
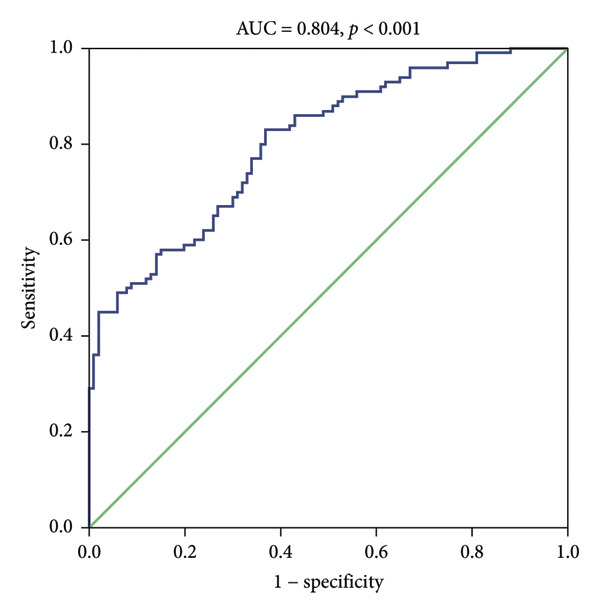
(b)

## 4. Discussion

The present study aimed to assess the potential association between serum miR‐126 levels and DN. In this study, we investigated 100 DN patients and found that the serum miR‐126 level significantly decreased in the DN group. Additionally, it also correlated with Hb1Ac and eGFR levels, which reflected the progression of DN, and both of the differences were statistically significant. Therefore, we suggested that miR‐126 could be a potential biomarker in the diagnosis of DN.

DN has been regarded as a major terminal complication of DM, which occurs in nearly 40% of T2DM patients. Moreover, there is a positive correlation between DN and the potential risk of terminal renal disease. Currently, the proper biomarkers are still being exploited to reflect DN from T2DM patients to minimize the disease progression in ESRD. Nowadays, there are therapeutic strategies for DN with the purpose of reducing its progression; however, most of them have turned out to be less effective [22]. Thus, there is an urgent need to explore novel biomarkers to facilitate early diagnosis and intervention in DN patients. Mounting research showed that the altered expression level of some microRNAs can be found in DN patients [23]. Since the pivotal role of microRNA in DN has received increasing attention, we aim to assess the diagnostic role of miR‐126 in DN.

The molecular mechanisms of microRNA action were complex and multidirectional in the development of DN. Recent studies have indicated that oxidative stress may play a vital role in the pathogenesis of T2DM [24, 25]. At high glucose levels, oxidative stress is induced through regulating glycolysis and advanced glycation end products, and oxidative stress also plays a pivotal role in the mechanism of DM and related complications [26]. Since several microRNAs have been reported to link to the development of DN, Akpınar et al. investigated the expression of 10 microRNAs in distinguishing 150 DN patients from the controls, suggesting that these miRNAs might be involved in the pathogenesis of DN [27]. Some studies demonstrated that diabetic groups with complications exhibited significantly higher miR‐21 and neutrophil gelatinase‐associated lipocalin (NGAL) and kidney injury molecule‐1 (KIM‐1) expression, which was linked to endothelial dysfunction and vascular remodeling [28, 29]. Also, previous data also showed a decrease of miR‐29c, which is related to increased inflammation and oxidative stress in T2DM patients [30].

Previous studies have demonstrated the beneficial effects of miR‐126 against oxidative stress, which can orchestrate endothelial progenitor cell functions under hypoxic conditions and inhibit ischemia‐induced oxidative stress and inflammation [15, 31]. Ahmed et al. reported that the expression of miR‐126, SPRED1, and PI3K/Akt phosphorylation was decreased in T2DM patients [32]. Moreover, another study suggested that miR‐126 affected the expression of IRS1, resulting in downregulated expression of PI3K/Akt pathway proteins, and also suppressed cell invasion and viability [33]. Furthermore, miR‐126 also regulated the downstream gene PIK3R2 in the PI3K/Akt pathway and promoted glomerular and diabetic wound repair in endothelial progenitor cells [34].

Collectively, the findings of this study may reflect the potential effect of oxidative stress, as reflected by circulating levels of miR‐126, in the pathophysiological mechanism resulting in renal damage in T2DM patients.

The study also had several limitations. First, this study included relatively small sample sizes, and all patients were recruited in a single center with an observational design, so the reliability and validity were limited. Second, this is a single‐center study that demonstrated an association between miR‐126 and the presence of DN, but it does not permit inference of causality, and its effect on the development of DN in the context of oxidative stress remains to be explored.

In conclusion, this study provides evidence to clarify that decreased levels of miR‐126 were linked to an increased susceptibility to developing DN compared with healthy volunteers. More importantly, the diagnostic role of miR‐126 remained significant in differentiating DN from T2DM patients. Based on the current data, it may help to realize that serum miR‐126 level was an independent determinant for the risk of the development of DN and also be regarded as a useful biomarker with independent diagnostic value in these patients. Nevertheless, additional studies in larger, multicenter cohorts will be required to elucidate the exact mechanism of miR‐126 in the pathogenesis of these patients, which may facilitate promoting the therapeutic applications of miR‐192 in predicting and treating DN.

## Ethics Statement

The study was approved by the Ethics Committee of the First Affiliated Hospital of Heilongjiang University of Chinese Medicine, and all participants signed the informed consent before enrollment.

## Conflicts of Interest

The authors declare no conflicts of interest.

## Author Contributions

Conception and design: Jie Yun and Yexu Song. Development of methodology: Tianqi Liu, Yake Lan, and Chaofan Dong. Acquisition of data: Xin Bao, Liming Zhu, and Shan Luo. Writing, review, and/or revision of the manuscript: Jie Yun, Liqun Song, and Yexu Song.

## Funding

This study was supported by the Postdoctoral Funding Project of Heilongjiang Province (Grant Number: LBH‐Q21043), Heilongjiang Provincial Natural Science Foundation of China (Grant Number: PL2024H215), Basic Research Support Plan for Excellent Young Teacher of Heilongjiang Province Provincial Undergraduate University (Grant Number: YQJH2023153), and Heilongjiang Administration of Traditional Chinese Medicine (Grant Number: ZHY2024‐222).

## Data Availability

The data that support the findings of this study are available on reasonable request from the corresponding author.
